# Formulation Approaches for Improving the Dissolution Behavior and Bioavailability of Tolvaptan Using SMEDDS

**DOI:** 10.3390/pharmaceutics14020415

**Published:** 2022-02-14

**Authors:** Jong-Hwa Lee, Gye-Won Lee

**Affiliations:** 1 Bioanalysis and Pharmacokinetic Research Group, Korea Institute of Toxicology, Daejeon 34114, Korea; jhl@kitox.re.kr; 2 Department of Pharmaceutics & Biotechnology, Konyang University, Daejeon 35365, Korea

**Keywords:** tolvaptan, SMEDDS, quality by design (QbD), bioavailability

## Abstract

Tolvaptan, a selective vasopressin receptor antagonist, is a Class IV agent of Biopharmaceutical Classification System (BCS). To improve bioavailability after oral administration, the new tolvaptan-loaded self-microemulsifying drug delivery system (SMEDDS) was further optimized using a “design of the experiment (DoE)” including components of D-optional mixture design. Based on a solubility study of tolvaptan in various oils, surfactants, and cosurfactants, Capryol^®^ 90, Tween 20, and Transcutol^®^ HP [or polyethylene glycol 200 (PEG 200)] were finally selected for optimization of tolvaptan-loaded SMEDDS formulations. The fitting models of, and poly-nominal equations for, all response variables were acceptable, as revealed by analysis of variance (ANOVA, *R*^2^ > 0.900, *p* < 0.0001). The optimized formulations A-1 (Capryol^®^ 90/Tween 20/Transcutol^®^ HP = 10%/70%/20% *w*/*w*) and B-1 (Capryol^®^ 90/Tween 20/PEG 200 = 10%/70%/20% *w*/*w*) with desirabilities of 0.905 and 1.000, respectively, showed low droplet size and the dissolution rate exceeded 95% at 15 and 60 min. The tolvaptan-loaded SMEDDS remained stable for 3 months under accelerated conditions, thus with no change in any of content, color, particle size, or dissolution rate. In a rat pharmacokinetic study, the bioavailability of formulations A-1 (16.6%) and B-1 (11.5%) were 23–33-fold higher than that of raw tolvaptan powder (0.5%). Thus, the use of “quality by design (QbD)” during development of tolvaptan-loaded SMEDDS improved the dissolution rate and oral drug bioavailability.

## 1. Introduction

Tolvaptan, the active ingredient in Otsuka’s Samsca^®^ tablets, is a selective vasopressin V2 receptor antagonist used to treat severe hyponatremia in patients with heart failure, cirrhosis, or syndrome of inappropriate secretion of antidiuretic hormone (SIADH) [1,2,3].

Tolvaptan is classified as a BCS Class IV drug with low solubility (50 ng/mL, 25 °C, pH 2–12) in aqueous solution and low permeability. The bioavailability was extremely low, 0.63% and 2% in rats and dogs, respectively, after oral dosing of power prepared using a jet-mill [4]. To improve the oral bioavailability, a pharmaceutical approach was used in terms of enhancement of solubility and dissolution rate, as well as permeability [5]. Various techniques such as liposomes, nanosuspensions, solid dispersions, and self-emulsifying formulations have been utilized to improve the solubility [6].

If drugs are poorly water-soluble, SMEDDS improves solubility, dissolution, and oral bioavailability. Many commercial SMEDDS preparations are commercially available, including Neoral^®^ (cyclosporine A), Fortovase^®^ (Saquinavir), and Agenerase^®^ (amprenavir) [7]. A SMEDDS is an isotropic and thermodynamically stable mixture of oil, surfactant, and drug cosurfactant [8,9]. The selection of a suitable self-emulsifying formulation depends upon the assessment of the solubility of the drug in various components, the area of the self-emulsifying region as obtained in the phase diagram, and the droplet size distribution of the subsequent self-emulsification [10]. In addition, quality by design (QbD) has been established to prepare a stable SMEDDS [11,12,13].

The purpose of this study was to develop and characterize the optimal SMEDDS formulation containing tolvaptan in order to improve its solubility and bioavailability. The formulations were evaluated for self-emulsification performance, droplet size, in-vitro drug release, stability, and bioavailability for use as an oral drug delivery system.

## 2. Materials and Methods

### 2.1. Materials

Tolvaptan (purity > 98.5%) was provided by Hwail Pharm (Pyeongtaek, Korea). Triethyl citrate, Peceol^TM^ (glyceryl monooleate, type 40), Maisin^®^ CC (glyceryl monolinoleate), Lauroglycol^®^ 90 (propylene glycol monolaurate type II), Lauroglycol^®^ FCC (propylene glycol monolaurate type I), Cremophor^®^ RH40 (PEG-40 hydrogenated castor oil), Capryol^®^ 90 (propylene glycol monocaprylate type II), Labrafil^®^ M 2125 CS (laouroyl polyoxyl-6-glecerides), Labrafil^®^ M 1944 CS (oleoyl polyoxyl-6-glycerides), Labrafac^®^ lipophile WL 1349 (medium-chain triglyceride), Labrasol^®^ (caprylocapryl polyoxyl-8-glycerides), and Transcutol^®^ HP (diethylene glycol monoethyl ether) were obtained from Gattefosse (Saint-Priest, France). Tween (polyethylene glycol sorbitan monolaurate) 20, Tween 40, Tween 80, polyethylene glycol (PEG) 200, PEG 400, castor oil, and oleic acid were purchased from Daejung (Seoul, Korea). Methanol and acetonitrile (HPLC grade) were purchased from Duksan (Seoul, Korea). All other chemicals and reagents were of analytical grade.

### 2.2. Solubility, and Construction of Pseudo-Ternary Phase Diagrams

The solubility of tolvaptan was tested in a variety of oils, surfactants, and cosurfactants. To a glass vial containing 3 mL of each excipient, an excess amount of tolvaptan was added. The samples were stirred with a magnetic stirrer and were orbitally shaken (250 rpm) in an incubator for 3 days at 37 ± 0.5 °C. The mixtures were centrifuged at 15,871 rcf for 5 min (Hanil, Daejeon, Korea), and supernatants were filtered using a PVDF membrane filter (0.45 μm, 13 mm, Whatman, Lawrence, KY, USA) prior to analysis using HPLC. The HPLC system (Shimadzu, Japan) consisted of an LC 20A pump, SPD-10 A VP variable spectrophotometric detector, and Capcell pak C18 column (5 μm, 4.6 × 150 mm, Shiseido, Japan). The mobile phase was composed of a mixture of acetonitrile, purified water, and phosphoric acid (47%/53%/0.1% *v/v*) at a flow rate of 1.0 mL/min, and the detection wavelength was set to 254 nm [14]. The calibration curve was obtained by plotting the area against tolvaptan concentration ranging from 0.625 to 40 μg/mL, using the following equation: *Y* = 48,819*X* − 381.26 (*r* = 0.9990). The intra-/inter-day precision (coefficient of variation < 0.8%) and accuracy (relative error < 1.6%) were within the acceptable limits.

To determine the concentrations of oil, surfactant, and cosurfactant, pseudo-ternary phase diagrams were constructed. Emulsification efficiency, droplet size, and polydispersity index (PDI) were measured to investigate the properties of the prepared SMEDDS. The selection criteria were 20–30 s of emulsification formation time (emulsification efficiency), <200 nm of droplet size and <0.3 of PDI.

### 2.3. Preparation and Optimization of Tolvaptan-Loaded SMEDDS

The amount of 30 mg of tolvaptan was dissolved in 1.0 g of a mixture of Capryol^®^ 90, Tween 20, and Transcutol^®^ HP (or PEG 200) to achieve final drug content (3%, *w/w*). After vortexing until a clear solution was obtained, the final mixture was placed at room temperature for 60 min.

The composition of the tolvaptan-loaded SMEDDS formulation was optimized using the D-optimal mixture design. The total amount of the components was kept constant in this design, including Capryol^®^ 90 (X1, oil phase), Tween 20 (X2, surfactant), and Transcutol^®^ HP or PEG 200 (X3, cosurfactant), while the proportions of the mixture components changed. The Capryol^®^ 90, Tween 20, and Transcutol^®^ HP (or PEG 200) concentrations were ranged from 10 to 30%, from 30 to 70%, and from 20 to 50%, respectively. The statistical experimental model was designed using Design-Expert software (V. 10.0; Stat-Ease Inc., Minneapolis, MN, USA). A total of 22 model formulations, including 10 estimate formulations, 7 estimate lack-of-fit formulations, 4 replicate formulations, and 1 additional center point formulation, were arranged randomly (Table 1 and Table 2). The dependent variables for determining the optimal SMEDDS formulation were the mean droplet size (Y1) and the percentage of drug dissolved at 15 min (Y2) and at 60 min (Y3). In Y1, Y2, and Y3, the numerical optimization criteria for SMEDDS were <250 nm, >90%, and >90%, respectively. A second-order polynomial equation with regression coefficients was used to fit the data. The significance and adequacy of the regression model were assessed using analysis of variance (ANOVA). The determination coefficient (*R*^2^) and the lack of fit were used to determine the adequacy of the response models. The interactions between each independent variable were also revealed using the polynomial equations, plots, and two-contour plots. After generating the polynomial equations for the dependent and independent variables, optimization of the dependent variables (Y1, Y2, and Y3) was performed using a desirability function to obtain the levels of X1, X2, and X3 that satisfied the dependent variable criteria [15].

### 2.4. Characterization of Tolvaptan-Loaded SMEDDS

Each formulation (100 mg) was diluted with distilled water (25 mL) and gently stirred. The droplet size distribution and Z-average diameter of tolvaptan-loaded SMEDDSs were measured using a Zetasizer Nano ZS (Malvern Instruments, Malvern, UK) at a scattering angle of 90° at room temperature using the dynamic light scattering (DLS) technique. Triplicates of each measurement were taken.

Transmission electron microscopy (TEM, Tecnai^TM^ G2 F30, FEI Company, Hillsboro, OR, USA) was used to examine the morphology of the microemulsion. After diluting tolvaptan-loaded SMEDDS 100 times with distilled water, the sample was stained for 5 min at 25 °C with a 2% phosphotungstic acid aqueous solution (PTA). The stained sample was then placed on a copper grid with one drop, and the sample was examined under the TEM after drying.

Using a USP type II (paddle) dissolution apparatus, the self-emulsification time of tolvaptan-loaded SMEDDS was determined. In a vessel with a paddle rotating at 50 rpm, 250 mL of simulated intestinal fluid (SIF, pH 6.8) was kept at 37 °C. A single amount of tolvaptan-loaded SMEDDS was added to each vessel. Self-emulsification time was defined as the time required to obtain a visually clear and transparent phase [12]. The HPLC method described in Section 2.2 was used to determine the content of tolvaptan.

### 2.5. In Vitro Dissolution Test

To evaluate the efficacy of the optimized formulation in alleviating tolvaptan’s poor aqueous solubility, the dissolution of tolvaptan from SMEDDS was carried out in four different dissolution media. The USP-NF 2021 type I dissolution apparatus was used to conduct an in vitro dissolution test using gelatin capsules (size 0) filled with SMEDDS containing 30 mg of tolvaptan (basket method). Using a dissolution tester (DRS-14, Labbindia, India) with agitation of basket at 50 rpm at 37 ± 0.5 °C in 900 mL of SIF (pH 6.8), the drug dissolution for the 22 experimental formulations was evaluated with 5 mL samples at 15 and 60 min. Finally, formulations A-1 (Capryol^®^ 90/Tween 20/Transcutol^®^ HP = 10%/70%/20% *w*/*w*) and B-1 (Capryol^®^ 90/Tween 20/PEG 200 = 10%/70%/20% *w*/*w*) were chosen as the optimal formulations. The optimized tolvaptan-loaded SMEDDS (formulation A-1 and B-1) was tested in distilled water, SGF (pH 1.2), acetate buffer (pH 4.0), and SIF (pH 6.8) to monitor how pH affected drug release. To maintain the sink condition, dissolution tests were performed in dissolution medium containing 0.22% sodium lauryl sulfate (SLS) [16]. At various time points (0, 10, 15, 30, 45, and 60 min), 5 mL samples were taken for analysis using HPLC, and the removed liquid was replaced with the same amount of fresh liquid.

To evaluate the similarity of release profiles for test formulations (formulations A-1 and B-1) and the reference (raw tolvaptan powder), the similarity factor, f_2_, was applied using the following equation [17,18]:

$$f_{2}=50\times \log\left\{{\left[1+\frac{1}{n}\overset{n}{\underset{t\;=1}{\sum }}{\left|R_{t}-T_{t}\right|}^{2}\right]}^{-0.5}\times 100\right\}$$

where R_t_ and T_t_ denote the cumulative drug dissolution of the reference and test formulations at the specified timepoint, respectively, and n denotes the number of sampling timepoints. When the f_2_ value is greater than 50, the drug release profiles of the reference and test formulations are considered to be similar [15].

### 2.6. Stability Test

The optimized tolvaptan-loaded SMEDDS was filled into hard gelatin capsules (size 0) and stored in a stability chamber (KCL-2000, EYELA; Tokyo Rikakikai Co., Ltd., Tokyo, Japan) for 3 months under intermediated conditions (25 °C/60% relative humidity (RH)) and accelerated degenerative conditions (40 °C/75% RH) [19]. The appearance, self-emulsifying properties, emulsion droplet size, drug content, and dissolution rate in SIF (pH 6.8) were all examined at different time intervals (0, 1, 2, and 3 months).

### 2.7. Pharmacokinetic Study

To investigate absorption, male Sprague–Dawley rats aged 7 weeks and weighing 195–219 g (Orient Bio, Seongnam, Korea) were given the optimized tolvaptan-loaded SMEDDS (formulations A-1 and B-1, 1 mL/kg) and raw tolvaptan powder (2 mL/kg) orally at a dose of 30 mg/kg. In addition, tolvaptan powder was given intravenously at a dose of 5 mg/kg (2 mL/kg) to monitor absolute bioavailability. Tolvaptan powder was solubilized in a mixture of 50% DMSO, 10% PEG 400, and 40% normal saline for intravenous injection at a volume of 2 mL/kg, and homogenized in 0.5% carboxymethyl cellulose for oral administration at a volume of 2 mL/kg. In four animals per group, blood samples (150 μL) were taken from the jugular vein at 0.25, 0.5, 1, 2, 4, 6, 8, and 24 h after oral dosing and at 0.083, 0.25, 0.5, 1, 2, 4, 6, 8, and 24 h after intravenous injection. The blood samples were centrifuged for 5 min at 17,600× *g*, and the separated plasma samples were kept at 70 °C until analysis. The animals were kept at a temperature of 20–26 °C, with a 12 h light–dark cycle and a relative humidity of 40–60% under the supervision of Chungnam National University’s Institutional Animal Care and Use Committee (202103A-CNU-053, Daejeon, Korea). Animals were fasted for 14 h prior to dosing and then given free access to water for another 4 h.

According to the established methods for tolvaptan in rat plasma [20,21], the bioanalytical method was optimized. The HP 1200 series system (Agilent Technologies, Santa Clara, CA, USA) was composed of a binary pump, degasser, autosampler, and column oven. An Xbridge BEH phenyl column (75 × 2.1 mm, 2.5 µm particle size; Waters, CA, USA) was used with the mobile phase consisting of (A) acetonitrile containing 0.1% formic acid and (B) water containing 0.1% formic acid with gradient elution (0 min: A 15%, 5 min: A 85%, 8 min: A 85%, 8.1 min: A 15%, 12 min: A 15%) at a 0.2 mL/min flow rate. Injection volume was 5 µL. The positive ion mode of the API 3200 Qtrap LC-MS/MS system (AB Sciex, Framingham, MA, USA) linked to HPLC was used. For tolvaptan and tolvaptan-D7, an internal standard, the ion source parameters were set as follows: ion spray voltage 5500 V, ion source temperature 550 °C, nebulizing gas 55 psi, and drying gas 55 psi. The MS parameters of declustering potential and collision energy were set to 46 V and 25 V, respectively. Multiple-reaction monitoring (MRM) was used to track the ion transition at *m*/*z* 449.15→252.20 for tolvaptan and *m*/*z* 456.15→259.20 for tolvaptan-D7. The Analyst software (version 1.4.2, AB Sciex) was used to operate LC–MS/MS and collect the data. The calibration curve was linear (weighting 1/*x*) from 2 to 5000 ng/mL with a correlation of 0.0999 (*Y* = 0.000854*X* − 0.000196).

The pharmacokinetic analysis was carried out by a noncompartmental analysis using Phoenix WinNonlin^®^ 8.1 (Pharsight Corp., Cary, NC, USA). The time (T_max_) to reach the peak concentration (C_max_) was obtained directly from the profile of the time-plasma concentration. The linear trapezoidal rule was applied to calculate the area under the plasma concentration-time curve from time zero to last quantification (AUC_last_), the area under the plasma concentration-time curve from time zero to infinity (AUC_inf_), the half-life (T_1/2_), elimination rate constant (K_el_), systemic clearance (CL), and the volume of distribution (V_d_) [22].

## 3. Results and Discussion

### 3.1. Solubility and Ternary Phase Diagram

**Figure 1:**
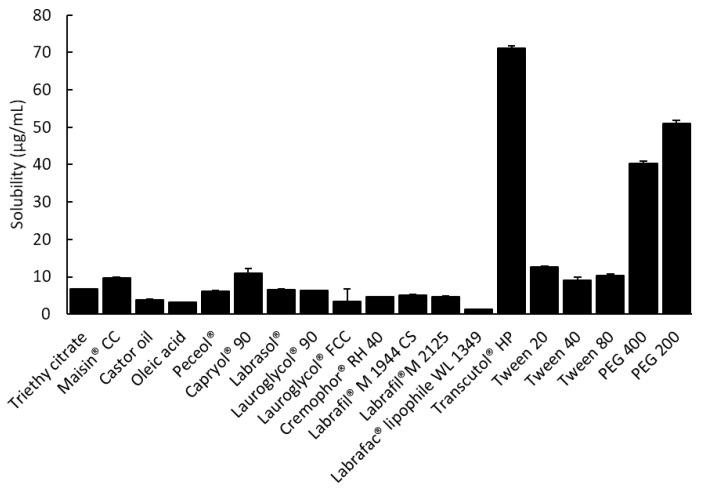
The solubility of tolvaptan in various excipients at 37 ± 0.5 °C (mean ± SD, *n* = 3).

The solubility of tolvaptan in various vehicles is presented in Figure 1. Tolvaptan showed high solubility in Capryol^®^ 90, Transcutol^®^ HP, PEG 200, and PEG 400 with 10.87 ± 1.24, 71.23 ± 0.62, 51.00 ± 0.75, and 40.30 ± 0.74 mg/g, respectively. The high solubility in Transcutol^®^ HP and PEG series is due to the ability of tolvaptan to form a hydrogen bond with the hydroxyl group [23]. Similarly, surfactants (Tween 20) composed of polyethyleneoxide groups showed high solubilization capacities (12.7 ± 0.24 mg/g) for tolvaptan. For the development of a SMEDDS formulation containing tolvaptan, Capryol^®^ 90, Tween 20, and Transcutol^®^ HP (or PEG 200) were chosen as the oil, surfactant, and cosurfactant, respectively, because each excipient had the highest solubility for tolvaptan.

**Figure 2:**
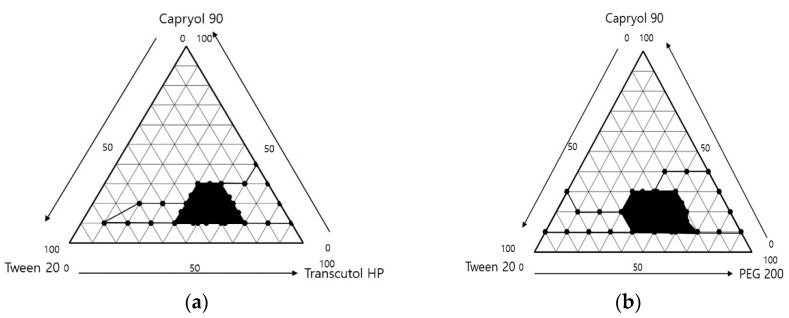
Ternary phase diagram of (**a**) Capryol^®^ 90/Tween 20/Transcutol^®^ HP and (**b**) Capryol^®^ 90/Tween 20/PEG 200. The black line indicates the transparent zone, and black area indicates the microemulsion region, with the prescription range indicating low PDI (<0.25) and droplet size (<250 nm).

**Figure 3:**
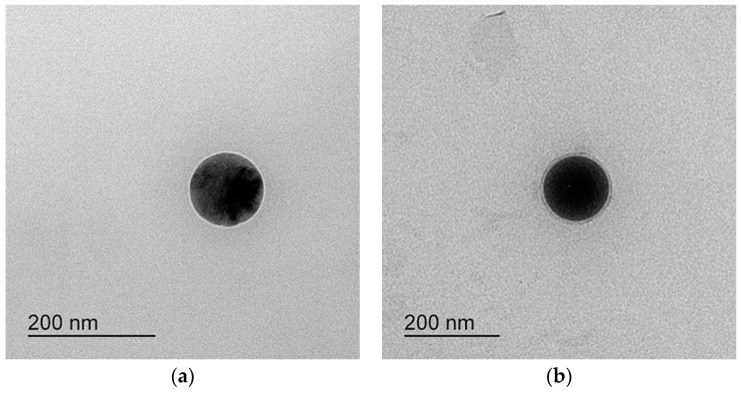
TEM of (**a**) formulation A-1 and (**b**) formulation B-1.

After dilution 250 times with distilled water, the results are shown in Figure 2. The area where the ternary mixture was showed with low droplet size under 250 nm was selected for further optimization studies. The 1 g ternary mixture composed of 0.1–0.3 g of Capryol^®^ 90 (oil), 0.4–0.7 g of Tween 20 (surfactant), and 0.1–0.3 g of Transcutol^®^ HP or PEG 200 (cosurfactant) revealed that an area could be used to optimize the SMEDDS formulation using the mixture design method. The TEM image of formulations A-1 and B-1 showed spherical globules (Figure 3).

### 3.2. Optimization by D-Optimal Mixture Design

**Figure 4:**
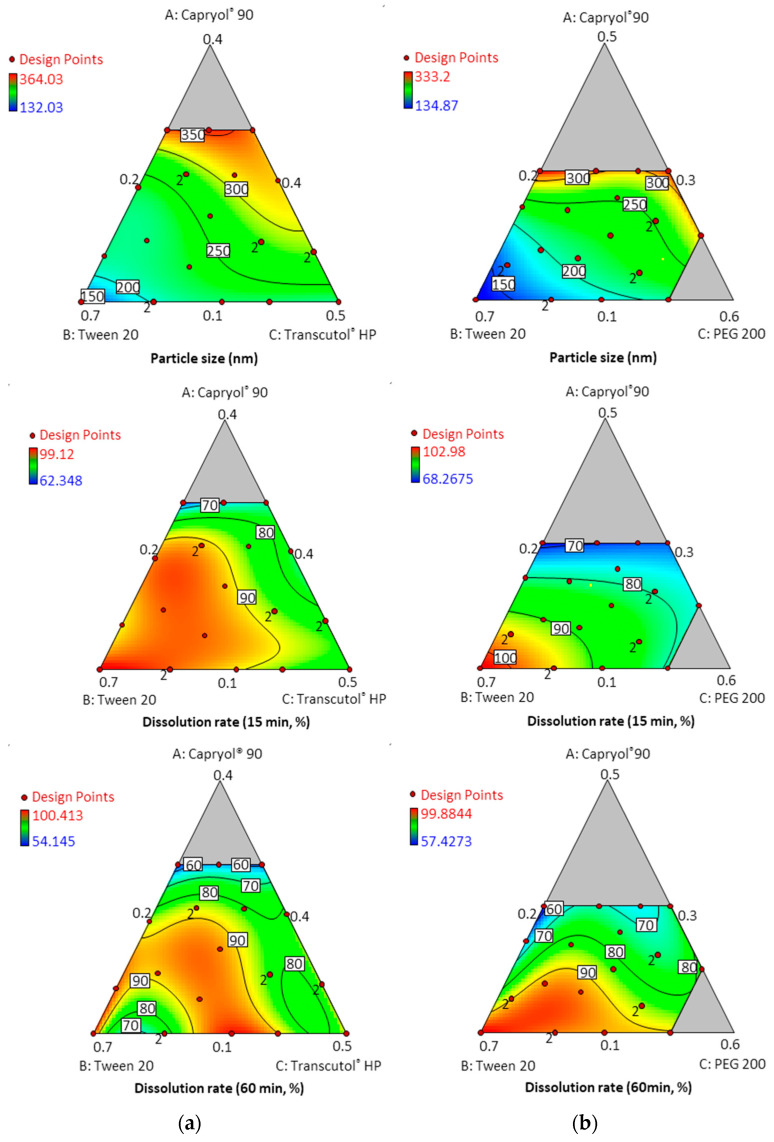
Contour plots for particle size and dissolution rate at timepoints of 15 min and 60 min; (**a**) Capryol^®^ 90 (X1)/Tween 20 (X2)/Transcutol^®^ HP (X3); (**b**) Capryol^®^ 90 (X1)/Tween 20 (X2)/PEG 200 (X3).

Table 1 and Table 2 show the results of each experimental run with independent variables and corresponding responses for the 22 formulations. In addition, Figure 4 depicts a 2D contour plot. The droplet size is a critical factor in the self-emulsification process because a smaller droplet size yields a larger interfacial surface area for drug absorption and allows for a faster rate of drug release, [24]. A aqueous dispersion in Capryol^®^ 90/Tween 20/PEG 200, and Capryol^®^ 90/Tween 20/Transcutol^®^ HP had mean droplet sizes of 235.36 ± 61.82 nm and 259.77 ± 53.64 nm, respectively.

The mean droplet size decreased as the amount of Tween 20 increased, and the size was located in the optimal region below a size of 250 nm. Overall average droplet size was smaller for PEG 200 than for Transcutol^®^ HP. Therefore, it is considered that these results can expect a higher dissolution rate.

The PDI, a droplet size distribution indicator, closest to zero indicates the most uniform droplet size [25]. The PDI of all SMEDDS formulations ranged from 0.098 to 0.40. In Table 2, formulation numbers 17 and 18 composed of Capryol^®^ 90/Tween 20/PEG 200 = 30%/41.3%/28.7% *w*/*w* and 30%/50%/20% *w*/*w*, respectively, showed a PDI value over 0.40 and the lowest dissolution rate.

**Figure 5:**
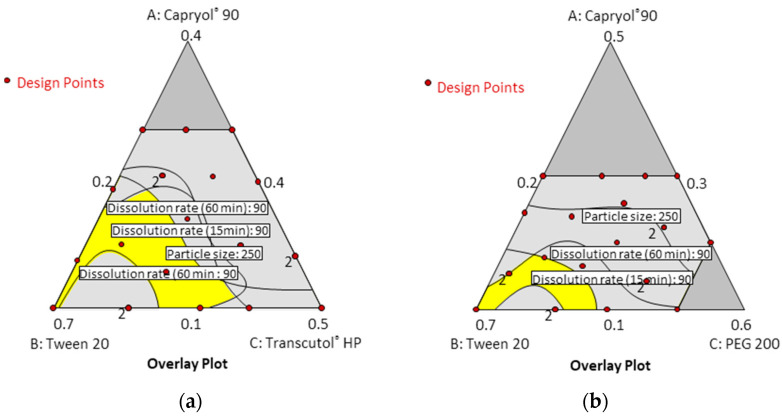
Overlay plot for the three response variables: (**a**) Capryol^®^ 90 (X1)/Tween 20 (X2)/Transcutol^®^ HP (X3); (**b**) Capryol^®^ 90 (X1)/Tween 20 (X2)/PEG 200 (X3).

In the dissolution tests for 22 formulations, the percentage of drug dissolved at 15 min and 60 min ranged from 54.15% to 100.14%. Formulations containing 30% Capryol^®^ 90 showed a low dissolution rate regardless of the type and content of cosurfactant. However, formulations containing 10% Capryol^®^ 90 maintained a high dissolution rate over 90% for 60 min. The 22 formulations simultaneously fit all of the responses observed using Design-Expert software. Table 3 shows the best-fitting models and polynomial equations for all response variables. The *R*^2^ values for all models were greater than 0.900, indicating that the generated polynomials fit the response data well (*p* < 0.0001 in all cases). Overlay plots were obtained by superimposing contour plots of independent variables, and the results are presented in Figure 5.

A numerical optimization technique with the desirability approach was used with various combinations of the independent variables to develop a new formulation with desired responses. The response variables are consequently closer to the largest value when the desirability value is close to 1 [26]. The experimental and predicted values (1.000–0.610) of the seven optimized formulations for each response are shown in Table 4. All formulations showed no more than 5% bias in the predicted results. In particular, formulations A-1 (Capryol^®^ 90/Tween 20/Transcutol^®^ HP = 10%/70%/20% *w*/*w*) and B-1 (Capryol^®^ 90/Tween 20/PEG 200 = 10%/70%/20% *w*/*w*) with desirabilities of 0.905 and 1.000, respectively, showed low droplet size and the dissolution rate exceeded 95% at 15 and 60 min. In addition, the formulations A-1 and B-1 were able to form clear and transparent microemulsions in less than 20 s upon adding distilled water and SIF (pH 6.8). Hence, formulations A-1 and B-1 were considered suitable for subsequent in vitro/in vivo study.

### 3.3. In Vitro Dissolution Study

**Figure 6:**
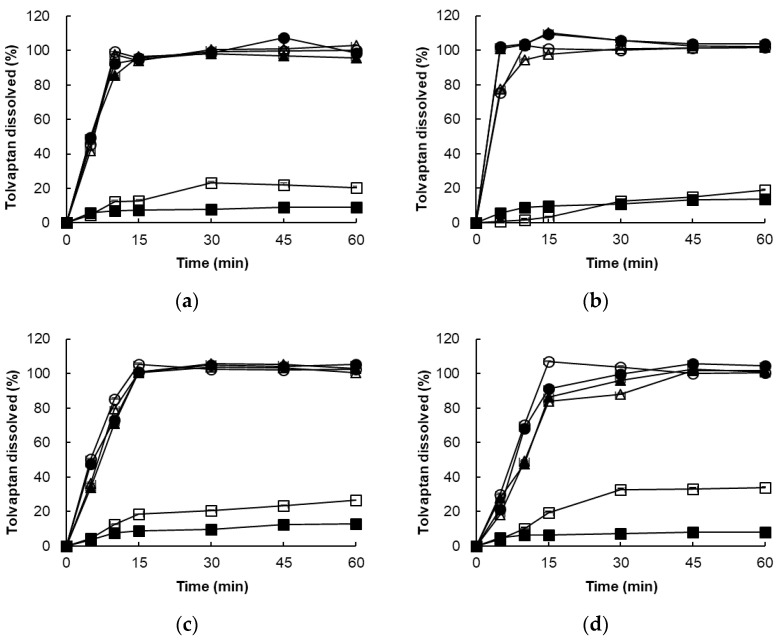
Dissolution profile of raw tolvaptan powder and formulations A-1 and B-1 in various media: (**a**) distilled water, (**b**) SGF (pH 1.2), (**c**) acetate buffer (pH 4.0), and (**d**) SIF (pH 6.8). ■ raw tolvaptan powder, □ raw tolvaptan powder plus 0.22% SLS, ● formulation A-1. ○ formulation A-1 plus 0.22% SLS, ▲ formulation B-1, △ formulation B-1 plus 0.22% SLS.

In four different dissolution media, the optimized tolvaptan-loaded SMEDDS formulations (formulation A-1 and B-1) showed a higher dissolution with approximately 90% in 60 min. On the other hand, raw tolvaptan powder showed low dissolution with only about 10–15% of the dose dissolved in 60 min (Figure 6).

In addition, formulations A-1 and B-1 showed a high dissolution rate in 60 min without being affected by the addition of 0.22% SLS, but the raw tolvaptan powder showed a dissolution rate of 20–30% in the 0.22% SLS medium. The rapid drug release from formulations A-1 and B-1 could be attributed to the spontaneous formation of a microemulsion with 130 nm small droplets, which provided a large surface area for drug release. The presence of surfactant and cosurfactant at the oil–water interface lowers surface tension, allowing oil droplets to release the drug only after coming into contact with the dissolution medium [27]. This indicated that formulations A-1 and B-1 successfully improved the solubility of tolvaptan regardless of the medium. In Table 5, the similarity factor f_2_ values of formulations A-1 and B-1 were greater than 50 regardless of pH, compared to the raw tolvaptan powder with 45.83 and 30.23 in distilled water and acetate buffer (pH 4.0) at 15 min and 60 min. As a result, the capacity for overall dissolution of the optimized tolvaptan-loaded SMEDDS formulations was considered unaffected by pH variations.

### 3.4. Stability

Under both intermediate and accelerated storage conditions, the drug contents and drug release at 60 min for formulations A-1 and B-1 were >95% under intermediate conditions (25 °C/60% RH over 3 months), and the mean particle sizes were 136.30 nm and 134.30 nm, respectively. The drug contents and drug release at 60 min were >95% and the mean particle sizes were 137.21 nm and 135.97 nm for the two formulations under accelerated conditions (40 °C/75% RH for 3 months). As a result, the emulsions did not change significantly over the course of 3 months. The formulations were compatible with hard gelatin capsules; there was no shell deformation, capsular degradation, or compromise of micro-emulsifying properties. There was no evidence of phase separation, drug precipitation, or capsule leakage.

### 3.5. Pharmacokinetic Study

**Figure 7:**
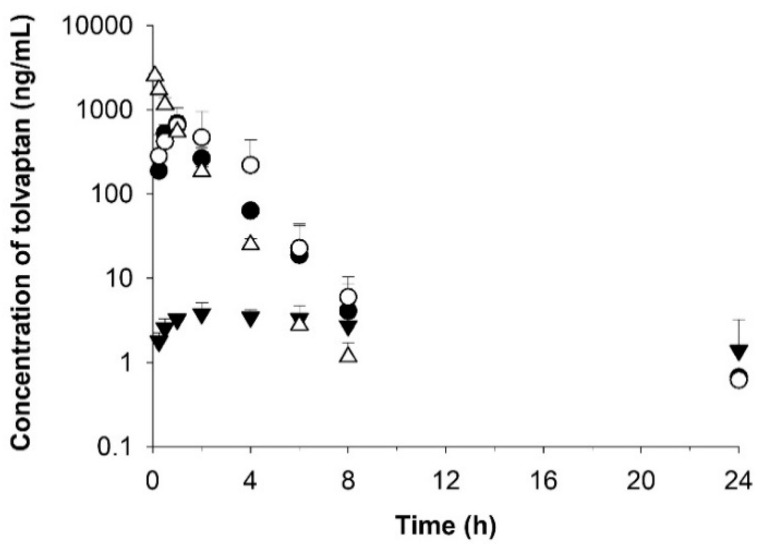
Temporal profiles of tolvaptan concentrations in rat plasma after oral administration of optimized formulations A-1 (○) and B-1 (●) and raw tolvaptan powder (▼) at a dose of 30 mg/kg and after intravenous administration of raw tolvaptan powder (△) at a dose of 5 mg/kg. Data are shown as the mean ± SD (*n* = 4).

Following an oral administration of formulations A-1 and B-1 at dose of 30 mg/kg as tolvaptan, the concentration rapidly increased and reached the peak concentration (C_max_) at 1.0 and 0.9 h (T_max_), respectively, with mean values of 686.6 and 660.2 ng/mL (Table 6 and Figure 7), compared to approximately 6 h in the tolvaptan powder-treated group, indicating that it is possible to increase the systemic exposure of drugs with poor bioavailability. Furthermore, C_max_ was 156-fold and 150-fold higher in the groups treated with formulations A-1 and B-1, respectively, than in the group treated the raw tolvaptan powder. The values of AUC_last_ for formulations A-1 and B-1 were 23-fold and 33-fold higher, respectively.

The half-life (T_1/2_) and elimination rate constant (K_el_) of the optimized formulations were similar in both groups with 4.5–5.7 h and 0.163–0.135 h, respectively. The clearance (CL) value in formulations A-1 and B-1 was approximately 4% that of the powder, and the V_d_ was 2–3% that of the raw tolvaptan powder. Considering the values of CL and V_d_, the optimized formulations (A-1 and B-1) were eliminated more slowly than the raw tolvaptan powder. 

The bioavailability of formulations A-1 and B-1 increased by 23- and 32-fold, respectively, compared to raw tolvaptan powder. According to the in vivo rat pharmacokinetic study, tolvaptan-loaded SMEDDS increased absorption and bioavailability by improving the solubility.

## 4. Conclusions

A tolvaptan-loaded SMEDDS was successfully prepared in this study using an optimized Capryol^®^ 90, Tween 20, and Transcutol^®^ HP (or PEG 200) composition. The in vitro drug dissolution study revealed relatively high dissolution in four media, with a cumulative drug release of approximately 90% in 60 min. The values of AUC and C_max_ for the optimized tolvaptan-loaded SMEDDS (formulations A-1 and B-1) were higher than those for raw tolvaptan powder in a rat pharmacokinetic study, indicating that the formulations had improved oral bioavailability.

## Figures and Tables

**Table 1:** Formulations from randomized runs in D-optimal mixture design (Capryol^®^ 90/Tween20/Transcutol^®^ HP) and observed responses (mean ± SD, *n* = 3).

Number	Variables (%)			Y1 (nm)	Y2 (%)	Y3 (%)	PDI
	X1	X2	X3				
1	10.0	40.0	50.0	218.73 ± 4.58	79.77 ± 0.43	89.64 ± 1.39	0.142
2	10.0	48.1	41.9	234.57 ± 1.55	84.24 ± 1.04	88.47 ± 0.79	0.129
3	10.0	53.6	36.4	206.27 ± 1.90	95.74 ± 0.83	100.41 ± 0.24	0.130
4 *	10.0	61.6	28.4	205.48 ± 8.99	95.78 ± 0.41	76.98 ± 0.05	0.146
5	10.0	70.0	20.0	132.83 ± 0.38	99.12 ± 0.91	98.85 ± 0.48	0.233
6	14.1	55.4	30.5	241.57 ± 0.55	90.34 ± 0.45	91.82 ± 1.08	0.185
7	15.4	64.7	20.0	221.90 ± 3.66	92.65 ± 1.65	94.95 ± 0.63	0.178
8 *	15.8	40.0	44.2	282.60 ± 9.39	82.27 ± 1.15	85.85 ± 1.24	0.190
9 *	17.0	45.5	37.5	286.85 ± 6.57	87.46 ± 0.17	84.07 ± 0.18	0.168
10	17.2	58.8	24.0	219.57 ± 2.94	94.78 ± 0.41	90.97 ± 0.53	0.209
11	20.0	50.0	30.0	246.50 ± 2.86	89.79 ± 0.29	94.41 ± 0.60	0.152
12	23.2	56.7	20.0	236.83 ± 1.88	93.30 ± 0.23	86.72 ± 1.19	0.189
13	24.2	40.0	35.8	324.07 ± 5.17	80.83 ± 0.60	81.30 ± 1.69	0.246
14	24.8	44.7	30.5	308.47 ± 8.33	80.34 ± 0.48	75.87 ± 1.40	0.223
15 *	24.9	50.3	24.8	279.62 ± 8.99	94.79 ± 0.16	88.49 ± 0.06	0.194
16	30.0	40.0	30.0	325.83 ± 3.96	73.02 ± 1.20	61.97 ± 2.24	0.366
17	30.0	45.1	24.9	364.03 ± 8.21	70.45 ± 2.21	59.57 ± 2.57	0.337
18	30.0	50.0	20.0	343.07 ± 4.50	62.35 ± 0.28	54.15 ± 0.57	0.394

X1: Capryol^®^ 90, X2: Tween 20, X3: Transcutol^®^ HP; Y1: mean particle size (nm); Y2: percentage dissolution at 15 min; Y3: percentage dissolution at 60 min; PDI: polydispersity index; * mean value expressed by duplicate measure.

**Table 2:** Formulations from randomized runs in D-optimal mixture design (Capryol^®^ 90/Tween20/PEG 200) and observed responses (mean ± SD, *n* = 3).

Number	Variables (%)			Y1 (nm)	Y2 (%)	Y3 (%)	PDI
	X1	X2	X3				
1	10.0	40.0	50.0	212.40 ± 2.59	79.21 ± 1.17	92.55 ± 0.88	0.168
2	10.0	50.5	39.5	162.20 ± 2.05	90.95 ± 4.37	94.74 ± 0.44	0.192
3 *	10.0	58.2	31.8	175.57 ± 7.59	95.31 ± 4.35	93.00 ± 4.85	0.255
4	10.0	70.0	20.0	134.87 ± 1.86	98.64 ± 0.35	99.22 ± 0.25	0.098
5 *	14.2	42.4	43.4	223.17 ± 10.39	82.45 ± 0.69	84.03 ± 2.21	0.184
6 *	15.4	62.4	22.2	157.94 ± 5.61	99.26 ± 5.27	92.59 ± 2.81	0.209
7	16.4	51.0	32.6	224.63 ± 3.38	87.47 ± 10.21	99.88 ± 1.27	0.168
8	17.7	56.0	26.3	190.97 ± 3.98	90.57 ± 0.50	89.79 ± 0.57	0.224
9	20.0	30.0	50.0	290.80 ± 9.86	80.62 ± 0.82	80.51 ± 0.73	0.257
10	20.0	44.0	36.0	250.17 ± 4.82	84.40 ± 0.50	78.28 ± 0.85	0.232
11 *	22.2	35.9	41.9	253.14 ± 5.85	77.18 ± 1.61	74.91 ± 4.51	0.315
12	23.9	48.8	27.3	242.40 ± 3.40	79.39 ± 0.95	83.46 ± 0.25	0.280
13	24.4	55.6	20.0	242.40 ± 3.69	77.57 ± 0.64	63.10 ± 0.65	0.245
14	25.9	40.0	34.1	220.97 ± 0.41	77.61 ± 0.92	74.65 ± 0.78	0.313
15	30.0	30.0	40.0	314.50 ± 11.44	71.51 ± 0.69	73.31 ± 1.01	0.368
16	30.0	34.8	35.2	328.43 ± 9.28	72.18 ± 0.67	71.19 ± 0.72	0.375
17	30.0	41.3	28.7	290.57 ± 10.57	70.19 ± 0.55	68.27 ± 0.38	0.472
18	30.0	50.0	20.0	333.20 ± 14.83	68.27 ± 0.76	57.43 ± 0.28	0.570

X1: Capryol^®^ 90, X2: Tween 20, X3: PEG 200; Y1: mean particle size (nm); Y2: percentage dissolution at 15 min; Y3: percentage dissolution at 60 min; PDI: polydispersity index; * mean value expressed by duplicate measure.

**Table 3:** Summary of the results of statistical analysis and model equations for measured response for D-optimal mixture design.

Models	Sequential *p*-Value	Lack of Fit *p*-Value	SD	*R* ^2^	Adjusted *R*^2^	Predicted *R*^2^
**Capryol** **^®^** **90/Tween 20/Transcutol^®^ HP**						
Y1 (nm): Mean droplet size						
RQuartic	<0.0001	0.3783	55.54	0.9766	0.9508	0.8317
Y2 (%): Dissolution rate at 15 min						
RQuartic	<0.0001	0.0016	9.55	0.9374	0.8905	0.7363
Y3 (%): Dissolution rate at 60 min						
RQuartic	<0.0001	0.0028	12.10	0.9798	0.9529	0.8073
Y1 = 444.41 × 1 + 132.87X2 + 219.07X3 − 142.82X1X2 − 54.61X1X3 + 184.27X2X3 − 176.88X1X2(X1 − X2) − 337.04X1X3(X1 − X3) + 89.74X2X3(X2 − X3) − 897.28X1X2^2^X3 + 832.07X1X2X3^2^ + 1064.45X1X2(X1 − X2)^2^						
Y2 = −145.82X1 + 99.95X2 + 79.72X3 + 457.81X1X2 + 445.03X1X3 + 7.11X2X3 + 346.48X1X2(X1 − X2) + 484.92X1X3(X1 − X3) − 1661.86X1^2^X2X3 + 402.25X1X3(X1 − X3)^2^						
Y3 = −66.48X1 + 98.59X2 + 89.66X3 + 259.96X1X2 + 276.11X1X3 + 15.96 + 124.15X1X2(X1 − X2) + 174.93X1X3(X1 − X3) − 101.34X2X3(X2 − X3) − 178.43X1^2^X2X3 + 477.40X1X2^2^X3 − 981.15X1X2X3^2^ − 351.94X2X3(X2 − X3)^2^						
**Capryol** **^®^** **90/Tween 20/PEG 200**						
Y1 (nm): Mean droplet size						
Special Quartic	<0.0001	0.0786	19.47	0.9341	0.8848	0.7339
Y2 (%): Dissolution rate at 15 min						
Quadratic	<0.0001	0.7250	3.08	0.9260	0.9029	0.8130
Y3 (%): Dissolution rate at 60 min						
RSpecial Quartic	<0.0001	0.5461	3.77	0.9334	0.9001	0.8456
Y1 = 1209.86X1 + 132.50X2 + 203.29X3 − 1354.83X1X2 − 1537.85X1X3 + 82.66X2X3 − 370.20X1X2(X1 − X2) − 1359.13X1X3(X1 − X3) − 34.91X2X3(X2 − X3) − 2847.10X1X2^2^X3						
Y2 = 12.54X1 + 103.37X2 + 69.13X3 + 41.09X1X2 + 121.03X1X3 + 8.25X2X3						
Y3 = 57.78X1 + 100.22X2 + 90.06X3 − 87.73X1X2 + 1.42X1X3 − 10.58X2X3 + 1279.55X1X2^2^X3 − 624.02X1X2X3^2^						

**Table 4:** Results of numerical optimization and evaluation of tolvaptan-loaded SMEDDS.

Formulation		A-1	A-2	A-3	A-4	B-1	B-2
Capryol 90^®^		10	19	19	11	10	14
Tween 20		70	57	55	40	70	36
Transcutol^®^ HP		20	24	35	49	-	-
PEG 200		-	-	-	-	20	50
Y1 (nm)	Predicted	132.89	221.79	221.96	230.16	132.5	241.98
	Observed	133.17 ± 1.27	228.35 ± 5.35	217.27 ± 3.35	228.83 ± 4.57	127.67 ± 0.64	243.87 ± 6.10
	Bias (%) *	0.23	2.96	−2.11	−0.56	−3.65	0.78
Y2 (%)	Predicted	99.95	96.39	91.66	82.36	103.37	79.21
	Observed	101.07 ± 0.23	96.50 ± 0.98	90.43 ± 0.44	83.22 ± 0.67	103.09 ± 0.6	76.69 ± 0.69
	Bias (%)	1.22	1.14	−1.34	1.04	−0.27	−3.18
Y3 (%)	Predicted	98.59	93.64	98.03	88.53	100.21	82.56
	Observed	99.85 ± 0.69	90.36 ± 0.77	96.99 ± 0.93	87.62 ± 0.64	100.93 ± 0.54	81.15 ± 0.54
	Bias (%)	1.27	−3.50	−1.06	−1.03	0.72	−1.71
Desirability		0.905	0.820	0.486	0.427	1.000	0.441

* (Observed value − predicted value)/predicted value × 100.

**Table 5:** The similarity factor (f_2_) of dissolution curves of raw tolvaptan powder and optimized formulations A-1 and B-1 between distilled water and SGF (pH 1.2), acetate buffer (pH 4.0), and SIF (pH 6.8) at 15 min and 60 min.

Formulation	f_2_					
	15 min			60 min		
	SGF (pH 1.2)	Acetate Buffer (pH 4.0)	SIF (pH 6.8)	SGF (pH 1.2)	Acetate Buffer (pH 4.0)	SIF (pH 6.8)
Raw Tolvaptan Powder	77.54	45.83	85.92	65.40	30.23	69.03
A-1	63.97	72.02	60.77	89.15	89.15	89.15
B-1	71.50	49.77	67.74	67.75	57.42	63.67

**Table 6:** Pharmacokinetic parameters of optimized tolvaptan-loaded SMEDDS (formulations A-1 and B-1) and raw tolvaptan powder in rats after intravenous administration of 5 mg/kg and oral administration of 30 mg/kg. Data are shown as the mean ± SD (*n* = 4).

Dosing Route	Formulation A-1	Formulation B-1	Reference (Raw Tolvaptan Powder)	
	Oral	Oral	Oral	Intravenous
C_max_ (ng/mL)	660.2 ± 402.6	686.6 ± 161.7	4.4 ± 0.9	-
AUC_last_ (h·ng/mL)	1962.4 ± 1603.9	1361.8 ± 233.3	58.7 ± 18.4	1967.8 ± 116.2
AUC_inf_ (h·ng/mL)	1966.4 ± 1604.3	1367.4 ± 232.6	57.5 ± 6.2	1969.0 ± 116.4
AUC_last_/dose	65.4 ± 53.5	45.4 ± 7.8	2.0 ± 0.6	393.6 ± 23.2
T_max_ (h)	0.9 ± 0.2	1.0 ± 0.0	6.0 ± 10.1	-
T_1/2_ (h)	4.54 ± 1.24	5.73 ± 1.75	7.73 ± 1.53	0.718 ± 0.036
V_d_ (mL/kg)	145,831 ± 89,352	184,045 ± 64,294	5,930,774 ± 1,576,551	2643.0 ± 273.9
CL (mL/h/kg)	20,952 ± 9221	22,430 ± 3607	526,005 ± 56,027	2546.5 ± 152.5
Kel (1/h)	0.163 ± 0.047	0.135 ± 0.057	0.093 ± 0.022	0.967 ± 0.047
Bioavailability (%)	16.6	11.5	0.5	-
